# Recovery among people with mental health challenges and alcohol and drug use issues in the Northern Territory, Australia

**DOI:** 10.1192/j.eurpsy.2024.270

**Published:** 2024-08-27

**Authors:** N. Tari-Keresztes, N. Armstrong, H. Gupta, J. A. Smith, S.-A. Endemann, S. Goding, J. Downes

**Affiliations:** ^1^Rural and Remote Health, Flinders University; ^2^Northern Territory Lived Experience Network, Darwin, Australia

## Abstract

**Introduction:**

The Northern Territory (NT) has Australia’s highest mental health burden. It has a diverse and transient population, including Aboriginal and Torres Strait Islander people and various multicultural communities. While peer support has been widely used nationwide, in the NT, peer support is poorly implemented in psychosocial support activities.

**Objectives:**

The NT Lived Experience Network (NTLEN), in allyship with a team of researchers from Flinders University, has secured multiple fundings aimed to develop, implement, and evaluate a peer education and recovery program called Recovery Together (RT) for individuals with mental health and alcohol and drug use issues and related challenges.

**Methods:**

The suitable evaluation approach was co-designed with live experience representatives from NTLEN and other local key stakeholders. It applied a mixed-method approach, including pre and post-program surveys (n=64) and individual interviews with program participants and the program delivery team (n=32). The evaluation findings were also informed by data collected by NTLEN via feedback forms n=38). We also used a co-design approach to develop survey instruments to ensure they were strengths-based and recovery-oriented.

**Results:**

Participants reported poor and fair self-perceived health, high stress levels, dissatisfaction with their relationships and relatively low recovery scores, which showed improvements at post-program completion. They discussed their journeys in the interviews and shared their experiences with local mental health services and the Recovery Together program. Many expressed that mental health professionals are not necessarily the care providers they feel comfortable engaging with. However, they described their experience with the peer program as highly positive, empowering, safe, non-judgmental, and beneficial, satisfying their support needs. The program gave them hope and tools to manage their mental health challenges and opportunities to gain insight into non-clinical aspects of recovery. Participants conceptualised personal recovery in their own words and described the facilitators and barriers to their recovery. They emphasised that recovery is being empowered, strong within themselves and the leader of their journey, living their best possible life, understanding themselves, having the necessary knowledge about mental health, and looking forward in a hopeful way.

**Conclusions:**

Our findings highlighted the demand and need for ongoing delivery of the RT program in the NT, which was highly effective in supporting personal recovery, addressing the service delivery gap and complementing the available clinical and mental health practices. They also showed the importance of providing recovery-oriented and trauma-informed education for medical and mental health professionals.

**Disclosure of Interest:**

None Declared

